# Supplementary material for: Wharton’s Jelly-Derived Mesenchymal Stem Cells Reduce Fibrosis in a Mouse Model of Duchenne Muscular Dystrophy by Upregulating microRNA 499
Source: Biomedicines. 2021 Aug 26;9(9):1089. doi: 10.3390/biomedicines9091089 (PMC8469349; doi:10.3390/biomedicines9091089)
Supplement: Supplementary file 1 [file biomedicines-09-01089-s001.zip › biomedicines-1339219-supplementary.pdf]

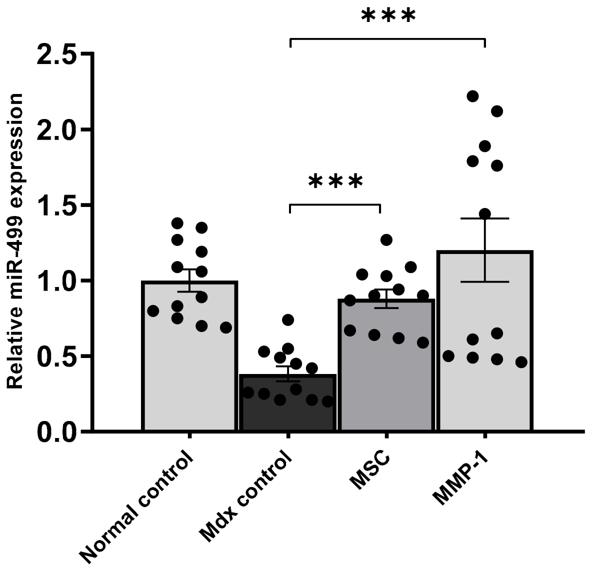

Figure S1. MMP-1 enhances miR-499-5p expression level in the gastrocnemius muscles of mdx mice. Relative miR-499-5p expression level was measured in skeletal muscles of each group. Administration of WJ-MSC and MMP-1 increased miRNA expression level in mdx mice. miR-26a-5p was used as an endogenous control; data are expressed as the mean ± SEM. Bars with different superscripts indicate significantly different values (***$p < 0.001$).

	Normal		Mdx	
	Human DNA (ng/100 ng of gDNA)	SEM	Human DNA (ng/100 ng of gDNA)	SEM
Gastrocnemius	0.01	0	2.73	0.59
Heart	15.00	12.14	2.29	1.09
Liver	109.49	22.66	48.82	23.57
Lung	4.43	1.71	37.42	15.09
Spleen	12.48	3.68	7.86	3.57

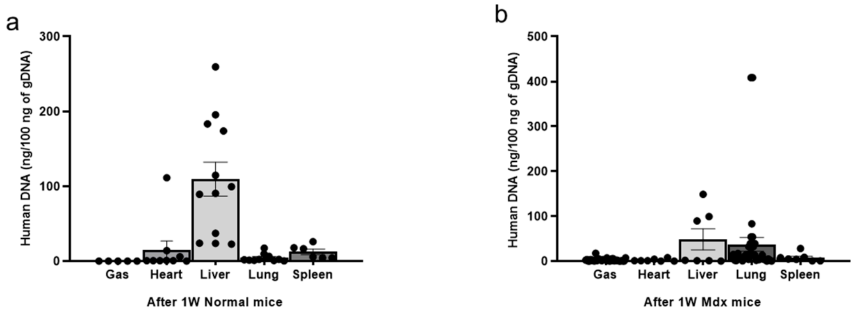

Figure S2. Quantitative analysis of Wharton's jelly-derived mesenchymal stem cells (WJ-MSCs) in various organs.

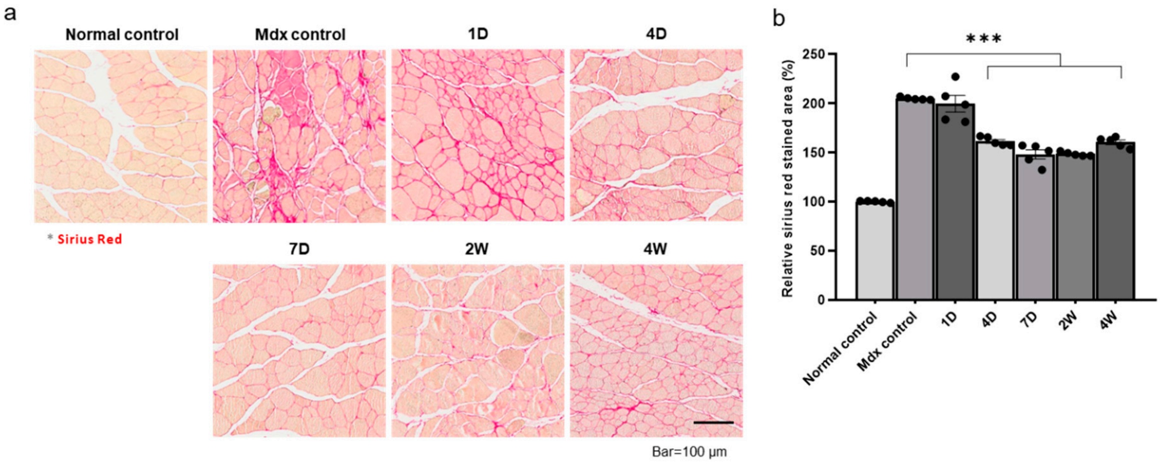

Figure S3. Duration of the anti-fibrotic effect of MSCs in the muscle. (a) Relative creatine kinase levels were measured in the serum from each group. **(b)** Representative images of immunohistochemistry for fibrosis detection. **(c)** Representative images of Sirius Red staining for measurement of fibrosis.

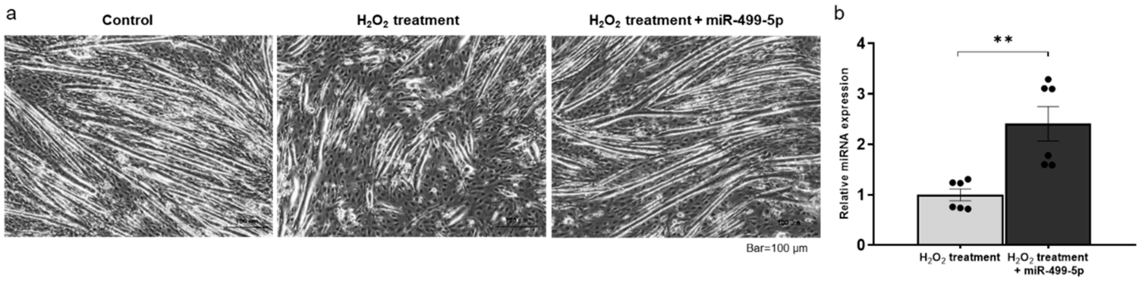

Figure S4. Efficiency of the miR-499-5p mimic in the H_2O_2-induced myotube fibrosis model. (a) Differentiated myotubes were treated with 2 mM H_2O_2 in the absence or presence of miR-499-5p mimic. Administration of miR-499-5p mimic induced recovery of myotubes damaged by H_2O_2 treatment (bar = 100 μm). **(b)** Relative miR-499-5p expression level was measured in H_2O_2-treated myotubes with or without miR-499-5p mimic. Administration of miR-499-5p mimic increased miRNA expression level. miR-26a-5p was used as an endogenous control; data are expressed as the mean ± SEM. Bars with different superscripts indicate significantly different values. (***p* < 0.01).
